# Downregulated Translation Initiation Signaling Predisposes Low-Birth-Weight Neonatal Pigs to Slower Rates of Muscle Protein Synthesis

**DOI:** 10.3389/fphys.2017.00482

**Published:** 2017-07-11

**Authors:** Ying Chen, Sydney R. McCauley, Sally E. Johnson, Robert P. Rhoads, Samer W. El-Kadi

**Affiliations:** Department of Animal and Poultry Sciences, Virginia Tech Blacksburg, VA, United States

**Keywords:** low-birth-weight, skeletal muscle growth, IGF-I, myostatin, translation initiation

## Abstract

Low-birth-weight (LBWT) neonates experience restricted muscle growth in their perinatal life. Our aim was to investigate the mechanisms that contribute to slower skeletal muscle growth of LBWT neonatal pigs. Twenty-four 1-day old male LBWT (816 ± 55 g) and normal-birth-weight (NBWT; 1,642 ± 55 g) littermates (*n* = 12) were euthanized to collect blood and longissimus dorsi (LD) muscle subsamples. Plasma glucose, insulin, and insulin-like growth factor-I (IGF-I) were lower in LBWT compared with NBWT pigs. Muscle *IGF-I* mRNA expression were lower in LBWT than NBWT pigs. However, IGF-I receptor mRNA and protein abundance was greater in LD of LBWT pigs. Abundance of myostatin and its receptors, and abundance and phosphorylation of smad3 were lower in LBWT LD by comparison with NBWT LD. Abundance of eukaryotic initiation factor (eIF) 4E binding protein 1 and mitogen-activated protein kinase-interacting kinases was lower in muscle of LBWT pigs compared with NBWT siblings, while eIF4E abundance and phosphorylation did not differ between the two groups. Furthermore, phosphorylation of ribosomal protein S6 kinase 1 (S6K1) was less in LBWT muscle, possibly due to lower eIF3e abundance. In addition, abundance and phosphorylation of eIF4G was reduced in LBWT pigs by comparison with NBWT littermates, suggesting translation initiation complex formation is compromised in muscle of LBWT pigs. In conclusion, diminished S6K1 activation and translation initiation signaling are likely the major contributors to impaired muscle growth in LBWT neonatal pigs. The upregulated *IGF-I R* expression and downregulated myostatin signaling seem to be compensatory responses for the reduction in protein synthesis signaling.

## Introduction

Insufficient placental growth, development, and function are suggested as primary causes of impaired fetal growth and development, collectively termed intrauterine growth retardation (IUGR), which contributes to low-birth-weight (LBWT) (Brown, 2014). In human infants, LBWT is often associated with increased risks of long-term metabolic diseases later in life (Barker et al., 2010; Gatford et al., 2010). Pigs exhibit the highest rate of LBWT (up to 20% of the litter) than any other domestic mammals, and develop LBWT spontaneously without any maternal nutrient or experimental interventions (Wu et al., 2006). The piglet serves as an infant model of LBWT due to metabolic and physiologic similarities with humans (Ferenc et al., 2014). More importantly, naturally occurring LBWT in pigs is characterized by asymmetric growth, the most prevalent feature (75%) in LBWT human infants (Bauer et al., 2003; Ferenc et al., 2014). A hallmark of asymmetric growth is the disproportionate slow growth of muscles compared to vital organs, such as the brain which is generally unaffected (Bauer et al., 2003; Rehfeldt and Kuhn, 2006). Given the large contribution of skeletal muscle to body mass and metabolic health, suppressed muscle development in LBWT neonates could be a major contributor to their impaired growth and lifelong metabolic disorders through adulthood (Brown, 2014).

Postnatal muscle growth is driven by two basic mechanisms (Rhoads et al., 2016). The first is through satellite cell-mediated myonuclear incorporation, which takes place via proliferation and fusion of satellite cells (Wozniak et al., 2005). The second is through the enlargement and elongation of existing muscle fibers as a result of muscle hypertrophy, which occurs when rates of protein synthesis surpass those of protein degradation (Glass, 2003; Miyazaki and Esser, 2009). We have previously shown that the satellite cell fusion is only modestly lower in muscle of LBWT neonatal pigs compared with their NBWT siblings suggesting that satellite cells are not intrinsically different between the two groups and is unlikely the major contributor to the impaired muscle growth of LBWT pigs (Chen et al., 2017). However, there is evidence that in skeletal muscle of newborn LBWT pigs, expression of enzymes involved in protein synthesis is decreased and enzymes associated with protein degradation are elevated (Wang et al., 2008). Our previous data, albeit from slightly older pigs (4 week), suggest that muscle protein synthesis is less in LBWT pigs compared with their NBWT littermates, while protein degradation is unaltered. Thus, the net result is less protein deposition and subsequent reduction of skeletal muscle mass in LBWT pigs (Zhu et al., 2015). However, the molecular mechanisms regulating protein synthesis in LBWT neonates have not been elucidated.

Protein synthesis is regulated by the dynamic interaction of signaling cascades either positively: insulin and insulin-like growth factor I-phosphoinositide 3 kinase-protein kinase B-mammalian target of rapamycin (INS-IGF-I-PI3K-PKB/Akt-mTOR) (Kimball et al., 2002; Ma and Blenis, 2009) and IGF-I-mitogen-activated protein kinase/extracellular signal-regulated kinase (MAPK/ERK) (Clemmons, 2009; Miyazaki and Esser, 2009), or negatively: myostatin (MSTN)-smad2/3 pathway (Han and Mitch, 2011; Schiaffino et al., 2013). Insulin and IGF-I exert their anabolic effects by binding to insulin receptor (INSR) and IGF-I receptor (IGF-I R) thereby initiating various intracellular kinase systems, including PI3K-PKB/Akt-mTOR and MAPK/ERK pathways (Czifra et al., 2006; Clemmons, 2009; Miyazaki and Esser, 2009). Activation of mTOR leads to the subsequent phosphorylation of the downstream target proteins, eukaryotic initiation factor (eIF) 4E binding protein 1 (4EBP1) and ribosomal protein S6 kinase 1 (S6K1), and triggers translation initiation which is the first and rate-limiting step in the process of protein synthesis (Davis and Fiorotto, 2009; Ma and Blenis, 2009; Laplante and Sabatini, 2012). In addition, MAPK/ERK pathway is necessary for IGF-I-induced muscle hypertrophy *in vivo* (Haddad and Adams, 2004) possibly through modulation of the MAPK-interacting kinases (MNKs), kinases responsible for direct phosphorylation and activation of eIF4E (Williamson et al., 2003; Egerman and Glass, 2014).

Myostatin, a member of transforming growth factor-β (TGF-β) superfamily, is expressed and secreted predominantly by skeletal muscle (Lee, 2004). MSTN binds to activin A receptor type IIB (ActRIIB), a type II TGF- β receptor, which results in activation of type I activin receptor-like kinase 4 (ALK4) or ALK5 (Elliott et al., 2012). Activated ALK5 phosphorylates intracellular mediators of signaling, smad2/3, resulting in inhibition of muscle growth (Han and Mitch, 2011) through a PKB/Akt-mTOR dependent signaling pathway (Lipina et al., 2010). Increased MSTN signaling coupled with reduced mTOR bioactivity may underline the deficits in muscle growth found in low weight weaned piglets reared by sows fed a low protein diet during gestation and lactation (Liu et al., 2015). How these signaling pathways influence protein synthesis in LBWT neonates is unclear.

In the present study, we profile the expression and activation of signaling pathways regulating protein synthesis in LBWT neonatal pigs. We show that low muscle IGF-I potentially counteracted the rise in IGF-I R expression since this resulted in no net effect on MAPK/ERK or PKB/Akt phosphorylation. Moreover, while MSTN-smad2/3 was less activated in LBWT pigs, this did not affect PKB/Akt phosphorylation, suggesting that the downregulated MSTN-samd2/3 signaling seems to be a compensatory mechanism. However, the reduced translation initiation signaling is likely what predisposes LBWT neonates to slower muscle growth rates.

## Materials and methods

### Animals and samples collection

All procedures involving animals were approved by the Virginia Tech Institutional Animal Care and Use Committee. Pregnant sows had free access to water and were fed daily a corn-soybean meal diet to meet NRC (2012) requirements for gestating sows. Piglets were weighed at birth and defined as normal-birth-weight (NBWT, 1,642 ± 55 g) when weight was within ± 0.5 *SD*, or low-birth-weight (LBWT, 816 ± 55 g) when weight was ≤ 2 *SD* of the litter average. Twelve pairs of 1-day old male pigs, one NBWT and one LBWT from the same litter, were used for the study. All pigs were fasted for 3 h before being euthanized for blood and muscle collection. Plasma was separated from heparinized blood by centrifugation at 1,200 × g in 4°C. Longissimus dorsi (LD) muscle samples (~2 cm) taken between the 12 and 15th rib were collected, snap frozen in liquid nitrogen and kept at −80°C until further analysis. LD muscles are composed by both type I and type II muscle fiber, mainly type IIB in pigs (Karlsson et al., 1993). In addition, LD muscles were chosen for sampling to provide adequate amount for analysis given the small body size of LBWT pigs.

### Plasma IGF-I, insulin, and glucose

Quantification of sample plasma IGF-I and insulin concentration was performed using human IGF-I and insulin ELISA kits according to manufacturer's instructions (R&D systems, Minneapolis, MN). Inter- and intra-assay coefficients of variation for the IGF-I assay were 8.0 and 4.2%, respectively. Inter- and intra-assay coefficients of variation for the insulin assay were 7.3 and 3.9%, respectively. Plasma glucose concentration was measured by GC-MS, as described early (El-Kadi et al., 2006).

### RNA extraction and quantitative real-time PCR

Skeletal muscle total RNA was extracted and purified using the Direct-zol RNA Miniprep Kit (ZYMO Research, Orange, CA). The concentrations of total RNA were measured with a spectrophotometer (NanoDrop, Thermo Fisher Scientific, Wilmington, DE). Genomic DNA contamination was removed by treatment with DNAse and total RNA (4,000 ng) was reverse transcribed into cDNA (High Capacity cDNA Reverse Kit, Applied Biosystems, Foster City, CA). Samples were mixed with Fast SYBR Green chemistry (Applied Biosystems) and gene-specific primers (Table 1) and added into 96-well plates in triplicates. Real-time quantitative PCR (qPCR) was performed on an ABI 7500 Fast Real-time PCR cycler (Applied Biosystems) to amplify samples for 40 cycles at 95°C for 23 s and 60°C for 30 s. Relative mRNA expression levels were calculated using the 2 ^−ΔΔCt^ comparative method and 18S abundance for normalization, which was not affected by birth weight.

**Table 1:** Nucleotide sequences of primers used for quantitative real-time PCR.

**Gene**	**Direction**	**Primer sequence**	**Accession No**.
*18 S*	Forward	5′- GTA ACC CGT TGA ACC CCA T -3′	AY265350
	Reverse	5′- CCA TCC AAT CGG TAG TAG CG -3′	
*IGF-I*	Forward	5′- GCA CAT CAC ATC CTC TTC GC -3′	NM_214256.1
	Reverse	5′- ACC CTG TGG GCT TGT TGA AA -3′	
*IGF-I receptor*	Forward	5′- CAT ACC AGG GCT TGT CCA AC -3′	NM_214172.1
	Reverse	5′- ATC AGC TCA AAC AGC ATG TCG -3′	
*Insulin receptor*	Forward	5′- GAA AGG GGG CAA GGG TCT AC -3′	XM_005654749.1
	Reverse	5′- CTC GGG TGC TTT GTT CTC CT -3′	
*IGF-II*	Forward	5′- GCT CGT CTT CTT GGC CTT G -3′	NM_213883.2
	Reverse	5′- CCG GCC TGC TGA AGT AGA A -3′	
*Myostatin*	Forward	5′- CCA GAG AGA TGA CAG CAG TGA TG-3′	NM_214435.2
	Reverse	5′- TTC CTT CCA CTT GCA TTA GAA GAT C-3′	
*ALK5*	Forward	5′- GGC AGA GCT GTG AAG CCT TA-3′	NM_001038639.1
	Reverse	5′- TGA TGC CTT CCT GCT GAC TG-3′	
*ActRIIB*	Forward	5′- GCA TCG CAA GCC TCC CTA T-3′	NM_001005350.1
	Reverse	5′- CTG TAG CAG GTT CTC GTG CTT C-3′	
*Decorin*	Forward	5′- ATC TCA GCT TTG AGG GCT CC-3′	NM_213920.1
	Reverse	5′- TGT CCA GAC CCA AAT CAG AAC AT-3′	
*Follistatin*	Forward	5′- CCC ATG TAA AGA AAC GTG CGA G-3′	NM_001003662.1
	Reverse	5′- TGC GGT AGG TTT TCC CAT CC-3′	

### Protein abundance phosphorylation

Abundance and phosphorylation status of signaling proteins was measured by western blot. Muscle samples were homogenized (1:7 w/v) in homogenization buffer, which contained 20 mM N-2-hydroxyethylpiperazine-N′-2-ethanesulfonic acid (HEPES), pH 7.4, 0.2 mM ethylenediaminetetraacetic acid, 2 mM ethylene glycol-bis (β-aminoethyl ether)-N,N,N′,N′-tetraacetic acid, 100 mM potassium chloride, 50 mM sodium fluoride, 50 mM β-glycerophosphate, and protease inhibitor cocktail (P8340, 1:200, Sigma, St. Louis, MO). Homogenized samples were centrifuged at 4°C to collect the supernatant. Muscle tissue lysates were diluted (1:1) in a 2 × SDS-PAGE sample buffer (Bio-Rad, Hercules, CA) and boiled for 5 min at 98°C.

Immunoblotting was performed by using specific primary antibodies listed in Table 2. Equal amounts (~25 μg) of total protein were electrophoretically separated through polyacrylamide gels. Denatured protein samples were run at the same time on triple-wide gels (CBS Scientific C, Del Mar, CA) to eliminate interassay variations. Following electrophoresis, proteins were transferred to a polyvinylidene difluoride membrane (Thermo Scientific). Blots were blocked with 5% bovine serum albumin (Research Products International Corp, Mount Prospect, IL) in tris-buffered saline with tween 20 (TBST) for 1.5 h at room temperature and then incubated with primary antibody overnight at 4°C. After extensive washing, blots were further incubated with peroxidase conjugated secondary antibodies (goat anti-rabbit or goat anti-mouse IgG-HRP conjugate; Bio-Rad) for 1 h at room temperature. Blots were washed and immune complexes were visualized by chemiluminescence (ECL, GE Healthcare, Piscataway, NJ) with a digital imager (BioDoc, Bio-Rad). Optical densitometry was performed using Image lab 4.0 (Bio-Rad). Total density values were normalized to the internal loading control (α-tubulin) and the phosphorylated to total protein ratios were determined. In addition, we verified that α-tubulin abundance was not affected by birth weight.

**Table 2:** Information of primary antibodies used for western blot.

**Antibody**	**Company**
ALK5	#AF3025; R&D systems, Minneapolis, MN
ActRIIB	#AF339; R&D systems, Minneapolis, MN
4EBP1	#A300-501A; Bethyl Laboratories, Montgomery, TX
eIF3e	#AB1114851; Abcam, Cambridge, MA
eIF4E	#9742; Cell Signaling Technology, Danvers, MA
eIF4G	#8701; Cell Signaling Technology, Danvers, MA
ERK1/2	#4695; Cell Signaling Technology, Danvers, MA
IGF-I receptor β	#9750; Cell Signaling Technology, Danvers, MA
MNK1	#2195; Cell Signaling Technology, Danvers, MA
MNK2	#sc-271559; Santa Cruz Biotechnology, Dallas, TX
Myostatin	#AB3239-I; Millipore, Temecula, CA
mTOR	#2983; Cell Signaling Technology, Danvers, MA
phospho-mTOR (Ser^2481^)	#2974; Cell Signaling Technology, Danvers, MA
p70 S6 kinase	#2708; Cell Signaling Technology, Danvers, MA
phospho-4EBP1 (Thr^46^)	#44-1170G; Invitrogen, Camarillo, CA
phospho-eIF4E (Ser^209^)	#9741; Cell Signaling Technology, Danvers, MA
phospho-ERK1/2 (Thr^202^/Tyr^204^)	#4370; Cell Signaling Technology, Danvers, MA
phospho-MNK1 (Thr^197/202^)	#2111; Cell Signaling Technology, Danvers, MA
phospho-PKB/Akt (Ser^473^)	#9271; Cell Signaling Technology, Danvers, MA
phosphor-eIF4G (Ser^1108^)	#2441; Cell Signaling Technology, Danvers, MA
phospho-S6K1 (Thr^389^)	#07-018-I; Millipore, Temecula, CA
phospho-smad2 (Ser^465/467^)/smad3 (Ser^423/425^)	#8828; Cell Signaling Technology, Danvers, MA
PKB/Akt	#9272; Cell Signaling Technology, Danvers, MA
Smad2/3	#8685; Cell Signaling Technology, Danvers, MA
α-Tubulin	#3873; Cell Signaling Technology, Danvers, MA

### Statistical analysis

Data were analyzed by PROC MIXED using SAS version 9.3 (SAS Inst. Inc., Cary, NC). For comparisons of the measurements in plasma and muscle tissue samples between LBWT and NBWT neonatal pigs, birth weight was the main effect and sow was the random effect. When a significant treatment effect was detected, means were compared using Tukey-Kramer Test. Data are expressed as the least square means ± SE and differences considered significance at *P* ≤ 0.05 unless otherwise noted.

## Results

### Plasma IGF-I, insulin, and glucose

**Figure 1:**
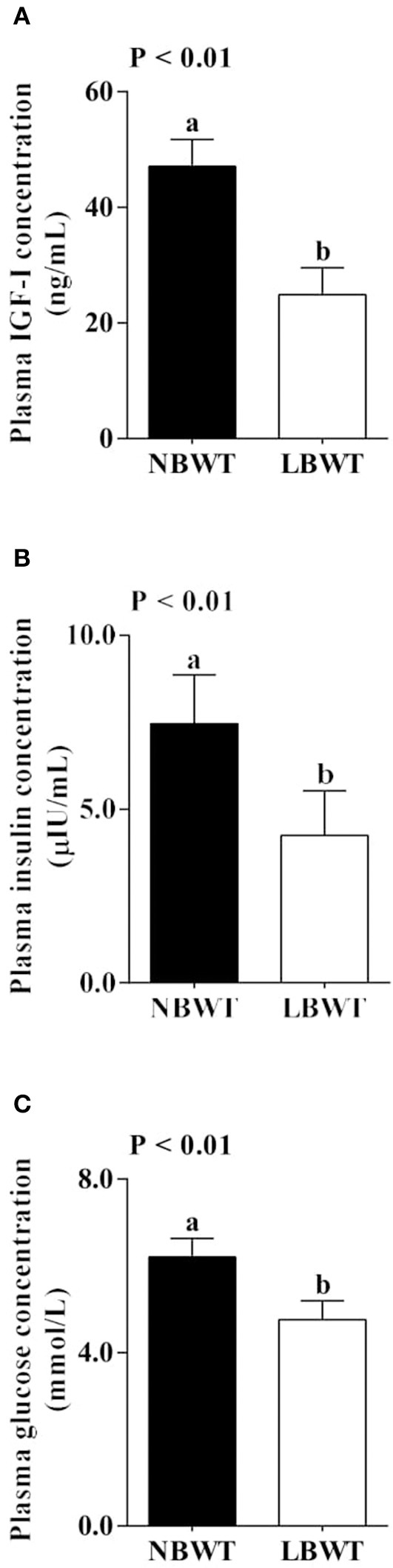
Plasma insulin-like growth factor (IGF)-I **(A)**, insulin **(B)**, and glucose **(C)** concentration in low-birth-weight (LBWT) and normal-birth-weight (NBWT) neonatal pigs. Results are means ± SE. *n* = 12. Values with different letters differ significantly (*P* ≤ 0.05).

Plasma IGF-I and insulin concentrations were 47 and 43% lower (*P* ≤ 0.05; Figures 1A,B) in LBWT compared with NBWT piglets. In addition, plasma glucose was 23% lower in LBWT than in NBWT pigs (*P* ≤ 0.05; Figure 1C).

### Expression of IGF system

**Figure 2:**
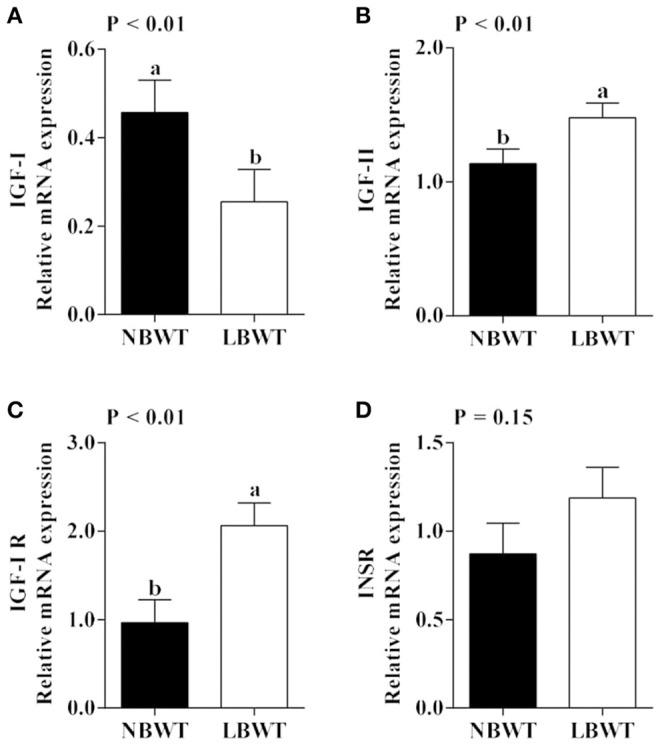
Gene expression of insulin-like growth factor (IGF) system in longissimus dorsi muscle of low-birth-weight (LBWT) and normal-birth-weight (NBWT) neonatal pigs. **(A**–**D)**: Relative mRNA expression of *IGF-I, IGF-II, IGF-I receptor* (*IGF-I R)*, and *insulin receptor* (*INSR)*. Results are means ± SE. *n* = 12. Values with different letters differ significantly (*P* ≤ 0.05).

**Figure 3:**
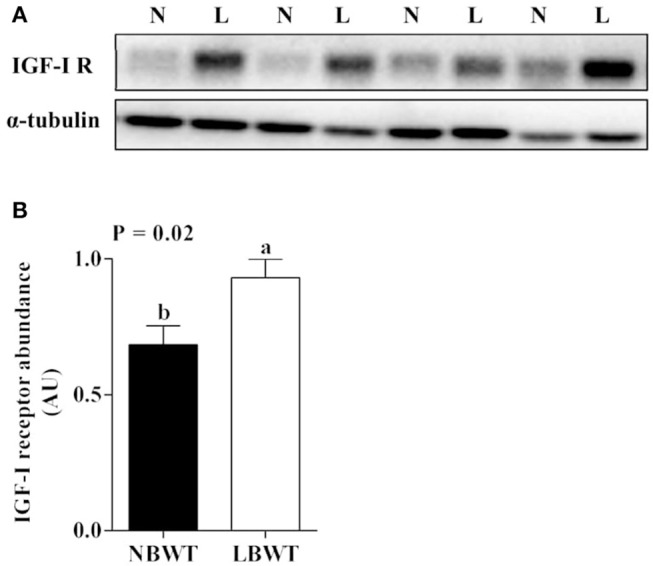
Protein abundance of IGF-I receptor (IGF-I R) in longissimus dorsi muscle of low-birth-weight (LBWT) and normal-birth-weight (NBWT) neonatal pigs. **(A)**: Representative western blot images from four pairs of NBWT and LBWT pigs (N, NBWT; L, LBWT). **(B)**: Abundance of IGF-I R was normalized to α-tubulin. Results are means ± SE. *n* = 12. Values with different letters differ significantly (*P* ≤ 0.05).

*IGF-I* mRNA expression was reduced in skeletal muscle of LBWT piglets by comparison with NBWT siblings (*P* ≤ 0.05; Figure 2A). By contrast, *IGF-II* and *IGF-I R* mRNA abundance was greater (*P* ≤ 0.05; Figures 2B,C) in LBWT than those in NBWT group. IGF-I R protein content was greater in LBWT LD compared with NBWT littermates (*P* ≤ 0.05; Figures 3A,B). No significant differences in mRNA expression of *INSR* in muscle between LBWT and NBWT pigs was noted (Figure 2D).

### PKB/Akt-mTOR signaling

**Figure 4:**
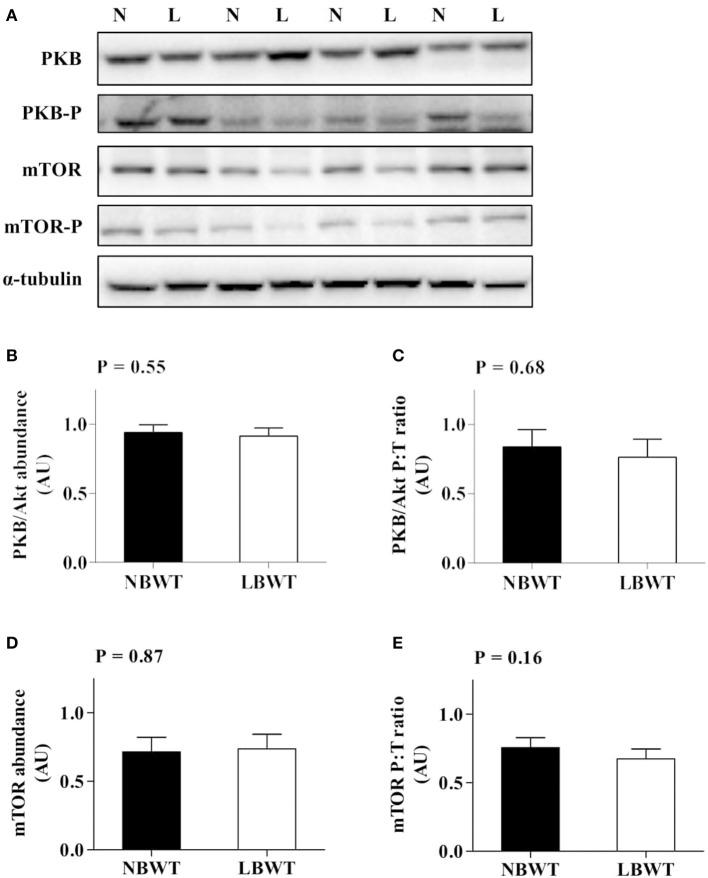
Protein abundance and phosphorylation of PKB/Akt and mTOR in longissimus dorsi muscle of low-birth-weight (LBWT) and normal-birth-weight (NBWT) neonatal pigs. **(A)**: Representative western blots from four pairs of LBWT and NBWT pigs (N, NBWT; L, LBWT). **(B**–**E)**: Protein abundance and phosphorylation of PKB/Akt and mTOR. Abundance was normalized to α-tubulin and phosphorylation normalized to the corresponding non-phospho-proteins. Results are means ± SE. *n* = 12.

**Figure 5:**
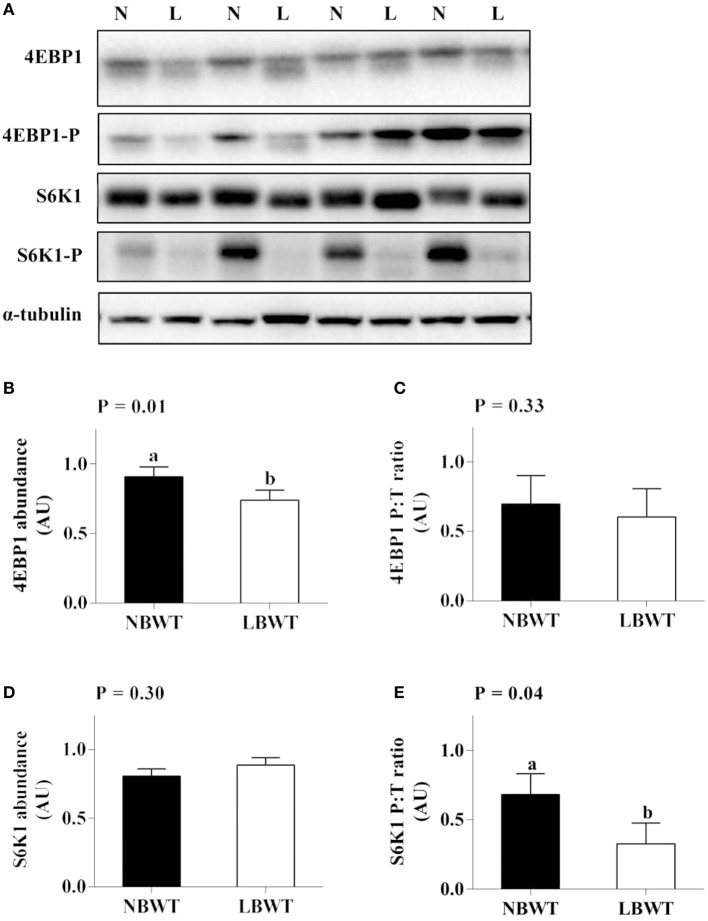
Protein abundance and phosphorylation of 4EBP1 and S6K1 in longissimus dorsi muscle of low-birth-weight (LBWT) and normal-birth-weight (NBWT) neonatal pigs. **(A)**: Representative western blots from four pairs of LBWT and NBWT pigs (N, NBWT; L, LBWT). **(B**–**E)**: Protein abundance and phosphorylation of 4EBP1 and S6K1. Abundance was normalized to α-tubulin and phosphorylation normalized to the corresponding non-phospho-proteins. Results are means ± SE. *n* = 12. Values with different letters differ significantly (*P* ≤ 0.05).

Neither the abundance nor the phosphorylation status of PKB/Akt in LD differed between LBWT and NBWT neonatal pigs (Figures 4A–C). In addition, there was no difference on protein abundance and phosphorylation of mTOR between two groups (Figures 4A,D,E). Although the 4EBP1 content was less (*P* ≤ 0.05) in LBWT LD lysates, the proportion of phosphorylated to total 4EBP1 was not different between the two groups (Figures 5A–C). Total S6K1 protein content was similar in both LBWT and NBWT LD lysates, however, phospho-S6K1 content was lower in LBWT lysates compared with NBWT counterparts (*P* ≤ 0.05; Figures 5A,D,E).

### MAPK/ERK signaling

**Figure 6:**
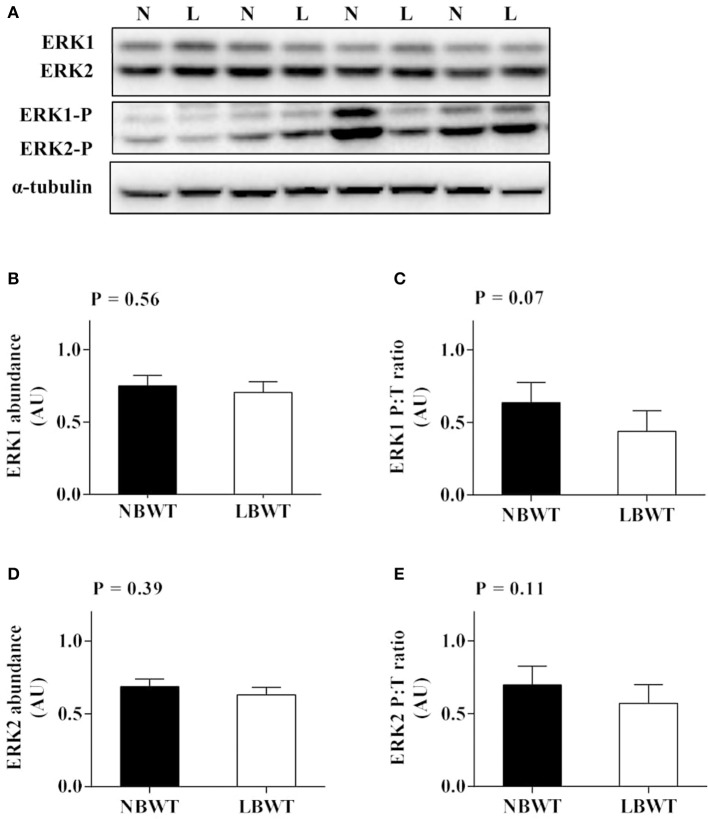
Protein abundance and phosphorylation of ERK1/2 in longissimus dorsi muscle of low-birth-weight (LBWT) and normal-birth-weight (NBWT) neonatal pigs. **(A)**: Representative western blots from four pairs of LBWT and NBWT pigs (N, NBWT; L, LBWT). **(B**–**E)**: Protein abundance and phosphorylation of ERK1 and ERK2. Abundance was normalized to α-tubulin and phosphorylation normalized to the corresponding non-phospho-proteins. Results are means ± SE. *n* = 12.

**Figure 7:**
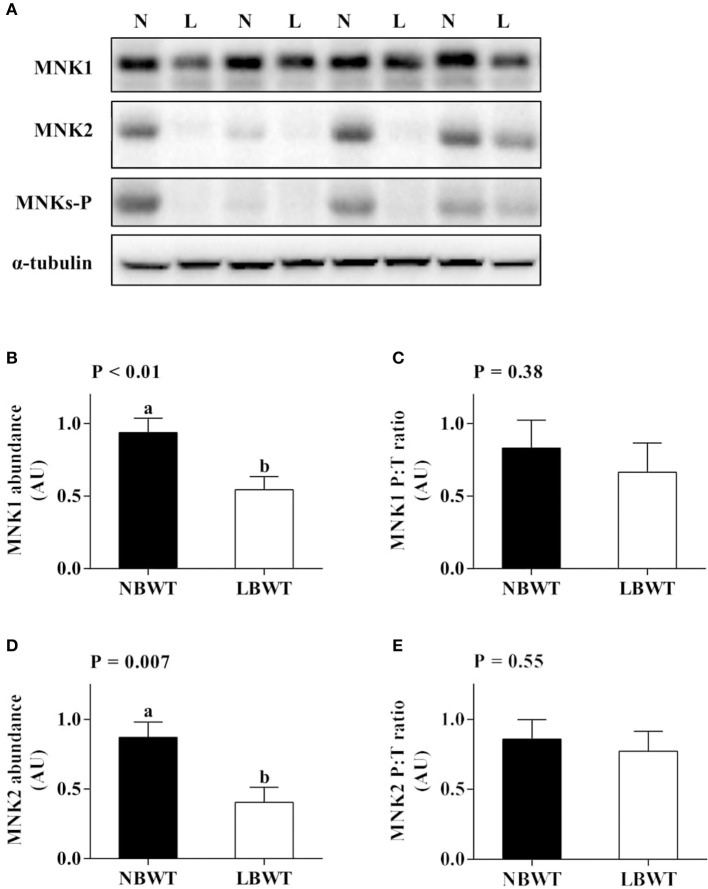
Protein abundance and phosphorylation of MNK1/2 in longissimus dorsi muscle of low-birth-weight (LBWT) and normal-birth-weight (NBWT) neonatal pigs. **(A)**: Representative western blots from four pairs of LBWT and NBWT pigs (N, NBWT; L, LBWT). **(B**–**E)**: Protein abundance and phosphorylation of MNK1 and MNK2. Abundance was normalized to α-tubulin and phosphorylation normalized to the corresponding non-phospho-proteins. Results are means ± SE. *n* = 12. Values with different letters differ significantly (*P* ≤ 0.05).

There were no differences in abundance of total ERK1/2 in muscle between LBWT and NBWT groups, while phosphorylation of ERK1/2 was modestly lower in LBWT pigs compared with their NBWT siblings, yet the differences were not significant (Figures 6A–E). Both MNK1 and MNK2 protein content were reduced (*P* ≤ 0.05) in LBWT muscle lysates by comparison with NBWT lysates, however, the calculated ratio of phosphorylated MNKs to total MNK1 and MNK2 revealed no significant difference between two groups suggesting equivalent bioactivity (Figures 7A–E).

### Translation initiation signaling

**Figure 8:**
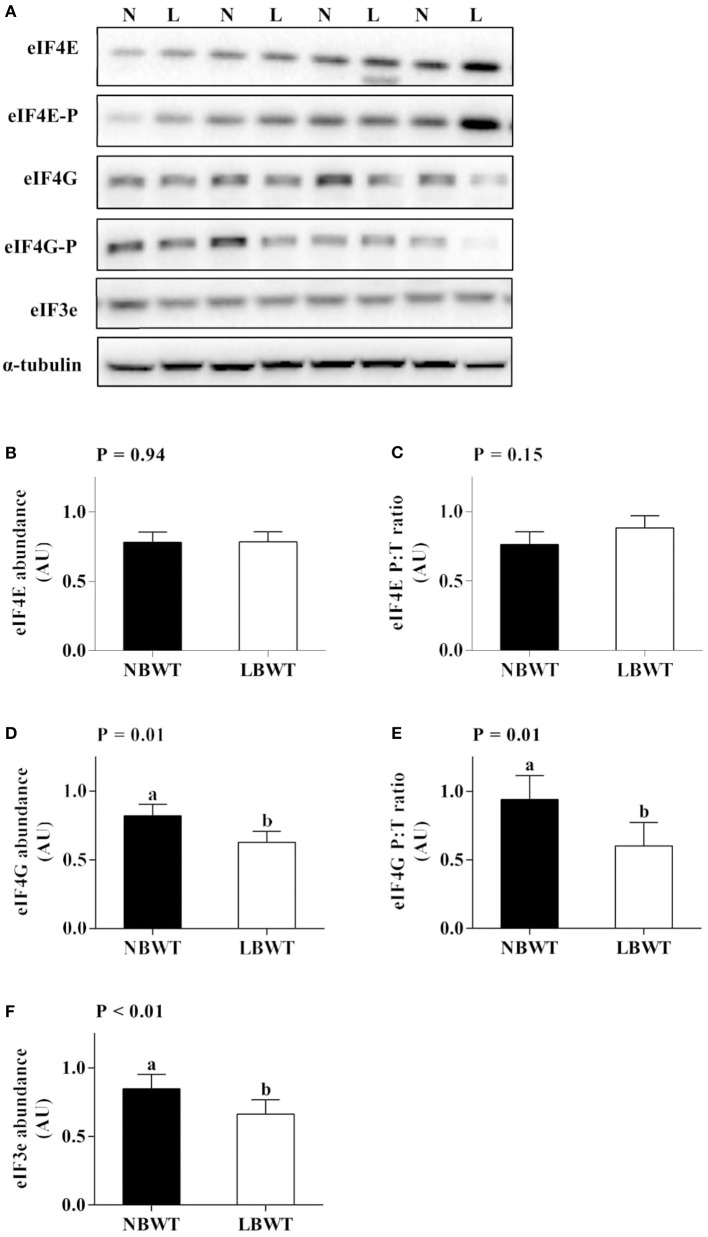
Translation initiation signaling in longissimus dorsi muscle of low-birth-weight (LBWT) and normal-birth-weight (NBWT) neonatal pigs. **(A)**: Representative western blots from four pairs of LBWT and NBWT pigs (N, NBWT; L, LBWT). **(B**–**F)**: Protein abundance and phosphorylation of eIF4E, eIF4G, and protein abundance of eIF3e. Abundance was normalized to α-tubulin and phosphorylation normalized to the corresponding non-phospho-proteins. Results are means ± SE. *n* = 12. Values with different letters differ significantly (*P* ≤ 0.05).

Abundance and phosphorylation of eIF4E were not different between LBWT and NBWT LD lysates (Figures 8A–C). However, abundance and phosphorylation of eIF4G were less (*P* ≤ 0.05) in LBWT muscle lysates compared with NBWT lysates (Figures 8A,D,E). In addition, abundance of eIF3e was less (*P* ≤ 0.05) in LBWT than NBWT muscle lysates (Figures 8A,F).

### MSTN signaling

**Figure 9:**
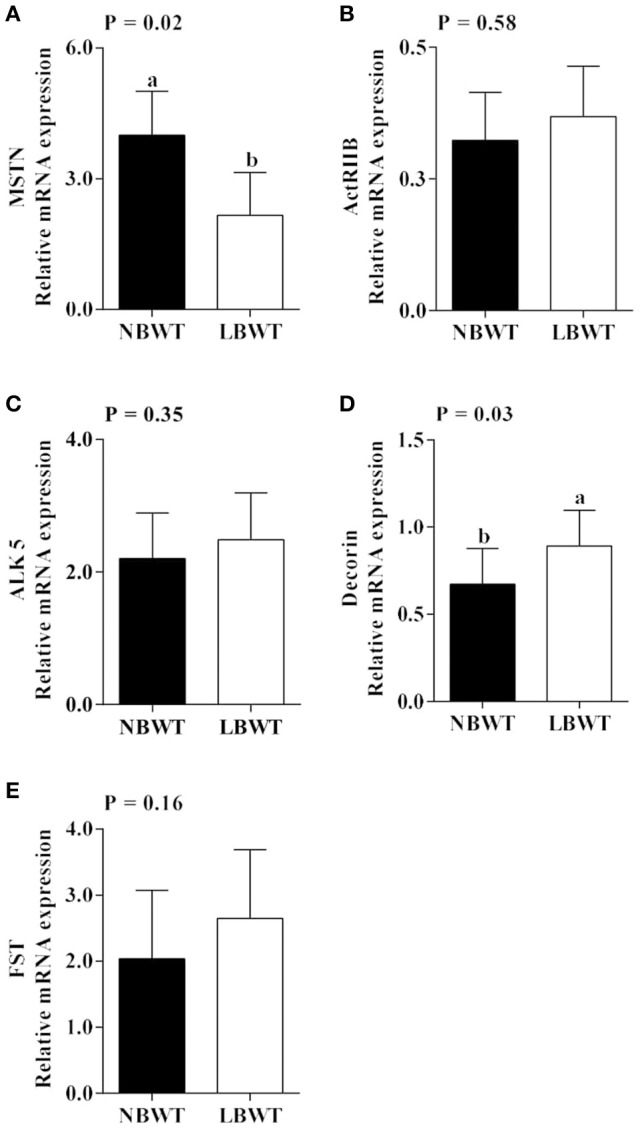
Gene expression of myostatin (MSTN) system in longissimus dorsi skeletal muscle from low-birth-weight (LBWT) and normal-birth-weight (NBWT) neonatal pigs. **(A**–**E)**: Relative mRNA expression of *MSTN, MSTN receptors, decorin, and follistatin (FST)*. Results are means ± SE. *n* = 12. Values with different letters differ significantly (*P* ≤ 0.05).

**Figure 10:**
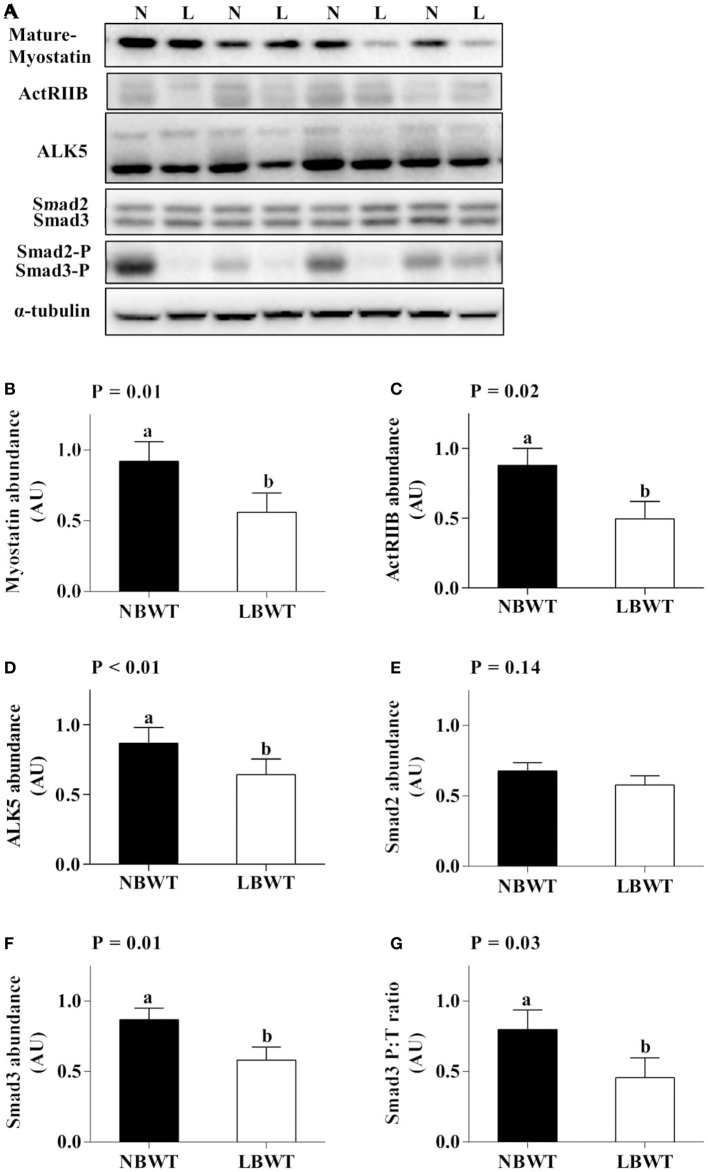
Myostatin signaling in longissimus dorsi muscle of low-birth-weight (LBWT) and normal-birth-weight (NBWT) neonatal pigs. **(A)**: Representative western blots from four pairs of LBWT and NBWT pigs (N, NBWT; L, LBWT). **(B**–**G)**: Protein abundance of myostain, ALK5 and ActRIIB, and protein abundance phosphorylation of smad2/3. Abundance was normalized to α-tubulin and phosphorylation normalized to the corresponding non-phospho-proteins. Results are means ± SE. *n* = 12. Values with different letters differ significantly (*P* ≤ 0.05).

Gene expression and protein abundance of MSTN was diminished in LBWT muscle lysates compared with NBWT lysates (*P* ≤ 0.05; Figures 9A, 10A,B). There were no differences in muscle mRNA expression of either MSTN receptors, *ALK5* and *ActRIIB*, or *follistatin* between LBWT and NBWT groups (Figures 9B,C,E). However, protein abundance of ALK5 and ActRIIB was lower in muscle of LBWT pigs compared with their NBWT siblings (Figures 10A,C,D). *Decorin* mRNA content was greater in LBWT than that in NBWT group (*P* ≤ 0.05; Figure 9D). Protein abundance of smad2 did not differ in LD muscle of LBWT from that in NBWT pigs (Figures 10A,E). However, abundance and phosphorylation of smad3 were lower in LBWT compared with their NBWT siblings (*P* ≤ 0.05; Figures 10A,F,G).

## Discussion

We profiled the expression and activation of signaling pathways that control skeletal muscle protein synthesis. The neonatal pig was used as a model for the human infant due to their similar anatomy, metabolism, and rapid growth rates in the neonatal period (Bauer et al., 2003; Ferenc et al., 2014). In addition, a comparison of LBWT and NBWT pigs from the same litter reduces possible variation due to confounding parental influence. The current study provides novel insights into putative molecular mechanisms contributing to the restricted skeletal muscle growth in LBWT neonatal pigs.

Low birth weight is associated with glucose intolerance, muscle insulin resistance and increased occurrence of type II diabetes later in life (Lindsay et al., 2000; Nobili et al., 2008). In the current study, plasma glucose concentration was lower in LBWT compared with NBWT neonatal pigs. The reduction in plasma glucose concentration has been previously observed in LBWT newborn pigs (Davis et al., 1997) and infants (Setia et al., 2006). The reduction in nutrient supplies before birth or limited gluconeogenic capacity associated with increased glucose utilization have been suggested as possible causes (Davis et al., 1997). In addition, our data suggested that plasma insulin concentration was lower in LBWT pigs compared with their NBWT littermates, a phenomenon also observed in LBWT infants (Setia et al., 2006).

It is well-established that the insulin and IGF system are a major regulators of prenatal and postnatal muscle growth mainly through protein metabolism signaling pathways (Florini, 1987; Duan et al., 2010). In this study, mRNA and protein abundance of IGF-I R were higher in skeletal muscle of LBWT compared with NBWT pigs. These data are in agreement with previous observations suggesting that the increase in IGF-I R expression starts during gestation and persists until birth in LBWT sheep (Muhlhausler et al., 2009) and pigs (Tilley et al., 2007). Elevated IGF-I R abundance would be expected to increase translation initiation signaling if IGF-I availability remains unchanged. However, our data indicated that while LBWT pigs had higher muscle IGF-I R abundance compared with NBWT siblings, plasma IGF-I concentration was lower and likely counteracted the rise in IGF-I R expression potentially negating any growth improvement. These data are consistent with observations that in LBWT infants (<1,500 g) (Kajantie et al., 2002), pigs (Davis et al., 1997), and rats (Fu et al., 2009), circulating IGF-I concentrations remain tightly correlated to growth in the perinatal period.

Circulating IGF-I is produced mainly by the liver in a growth hormone-dependent manner during postnatal life (Puche and Castilla-Cortazar, 2012). However, locally produced rather than hepatic IGF-I appears to have a greater effect on postnatal muscle growth (Yakar et al., 1999). This stems from observations that the highest *IGF-I* mRNA expression occurs in neonatal pig muscle which coincides with periods of fastest growth rates and this expression falls later in life (Gerrard et al., 1998). Conversely, circulating levels of IGF-I increase soon after birth and peak in adults (Puche and Castilla-Cortazar, 2012). In the current study, reduced muscle *IGF-I* expression may further negatively affect muscle growth in LBWT neonatal pigs.

Insulin and IGF-I activate two cascades that control protein synthesis signaling: PI3K-PKB/Akt-mTOR and MAPK/ERK pathways (Clemmons, 2009). The regulation of protein synthesis by mTOR occurs through, 4EBP1 and S6K1, two downstream effectors that tightly regulate translation initiation (Carrera, 2004; Gingras et al., 2004). Our data suggest that 4EBP1 protein abundance was lower in LBWT compared with that of NBWT pigs, however the proportion of phosphorylated to total 4EBP1 was not different between the two groups. The lower abundance of 4EBP1 may lead to an increase in translation initiation by dissociating from eIF4E (Wilson et al., 2009). In practice, however, this may not have been the case since abundance and phosphorylation of eIF4E were not different between the two groups. This would suggest that under the current experimental conditions, the downregulation of 4EBP1 expression was a compensatory response. Alternatively, 4EBP1 expression was not low enough to cause a response on availability of eIF4E for translation initiation in LBWT neonatal pigs.

S6K1 is the other major mTOR substrate. Compared with wild-type mice, S6K1^−/−^ mice are ~30% smaller in size at the early embryonic stage and remain nearly 15% smaller than normal as adults (Klammt et al., 2008). In the current study, there was no difference in S6K1 abundance between the two groups. However, S6K1 phosphorylation was lower in muscle of LBWT pigs compared with their NBWT littermates. The activation of S6K1 occurs through mTOR/raptor-mediated phosphorylation on Thr^389^ (Kim et al., 2002). Upon activation, mTOR/raptor is recruited to eIF3, which is a scaffold protein involved in the phosphorylation of S6K1 (Holz et al., 2005). Our data suggested that abundance of eIF3e (a subunit of eIF3) was lower in muscle of LBWT than NBWT pigs, which would explain the reduction in S6K1 phosphorylation.

The second signaling cascade activated by IGF-I is MAPK/ERK pathway. In the current study, abundance and phosphorylation of ERK1/2 in muscle did not differ between LBWT and their NBWT siblings indicating that signaling through this pathway is not a contributor to the reduction in translation initiation signaling observed in LBWT pigs. The association of the ERK pathway with translation initiation occurs through MNKs (Williamson et al., 2003; Egerman and Glass, 2014). The two kinases MNK1 and MNK2 regulate translation initiation by phosphorylating eIF4E at Ser^209^ (Ueda et al., 2004). In the current study, phosphorylation of MNKs did not differ between the two groups, which is consistent with the lack of differential ERK1/2 activation between LBWT and NBWT pigs. However, the abundance of MNK1 and MNK2 was lower in muscle of LBWT pigs in comparison with NBWT littermates. Furthermore, eIF3e is required for the recruitment of MNKs and phosphorylation of eIF4E (Walsh and Mohr, 2014). Although MNKs and eIF3e abundance was lower in muscle of LBWT compared with NBWT pigs, the lack of a concomitant reduction in eIF4E phosphorylation, while unexpected, suggested that eIF4E was not limiting translation initiation in LBWT pigs.

MNKs do not form a stable binary complex with eIF4E instead, the two proteins initially bind to the scaffolding protein eIF4G, which localizes the kinase and substrate into physical proximity (Pyronnet et al., 1999). In the current study, abundance and phosphorylation of eIF4G were significantly lower in muscle of LBWT than NBWT pigs, indicating that formation of translation initiation complex may be negatively affected. There is growing evidence to suggest that higher eIF4E phosphorylation does not increase the rate of protein synthesis, but assembly of the active eIF4E-eIF4G complex is the main mechanism controlling translation initiation (Scheper and Proud, 2002). Moreover, eIF4G phosphorylation at Ser^1108^ is a more appropriate molecular maker associated with enhanced protein translation, given that phosphorylation at this site is tightly correlated with protein synthesis (Vary and Lynch, 2006; Vary et al., 2006). We are currently investigating the significance of the reduction in MNKs and eIF4G expression on translation initiation complex formation in LBWT pigs.

In addition to anabolic stimuli, protein synthesis is inhibited by MSTN. Our data suggested that mRNA and protein expression of MSTN were lower in muscle of LBWT pigs compared with their NBWT littermates. This is in contrast to a previous study in which mRNA expression of *MSTN* was 65% higher in LD muscle of LBWT compared with NBWT newborn pigs (Ji et al., 1998). Although the basis for this discrepancy remains unclear, sex differences of the pigs between studies may be responsible for some of the observed differences and may relate to sexual dimorphism.

The activity of MSTN is regulated by follistatin (FST) (Barbe et al., 2015) and decorin (Miura et al., 2006), binding proteins that prevent MSTN inhibitory effect on skeletal muscle growth. Overexpressing decorin *in vitro* enhances myoblast differentiation rate by upregulating FST and downregulating MSTN expression (Li et al., 2007). In the current study, while there was no difference in *FST* mRNA expression, *decorin* expression was higher in muscle of LBWT compared with NBWT pigs. Accordingly, more MSTN may be sequestered by decorin and reduce its inhibitory action on protein synthesis signaling. In addition, reduced abundance of MSTN receptor, ALK5 and ActRIIB, suggested lower MSTN binding and may further decrease the activation of downstream signaling pathway. Indeed, protein abundance and phosphorylation of smad3 were lower in LBWT pigs compared with their NBWT littermates indicating a reduction in MSTN signaling and would be expected to promote protein synthesis signaling. However, this is unlikely since skeletal muscle weight from birth (Wang et al., 2008) until 21 days (Zhu et al., 2015) remains lower (~50%) in LBWT by comparison with NBWT pigs.

It is believed that MSTN's inhibition of muscle growth is directly mediated by locally produced MSTN and possibly by the IGF system. The expression of IGF-I R is higher in MSTN null neonatal mice skeletal muscle (Williams et al., 2011). Our data suggest that the higher expression of IGF-I R in muscle of LBWT pigs may be associated with decreased MSTN abundance in these pigs compared with their NBWT siblings. In addition, inhibition of smad2/3 promotes muscle hypertrophy via mTOR and overexpression of activated PKB/Akt completely prevents the atrophic effect of MSTN signaling (Sartori et al., 2009). However, in the current study there was no difference in abundance or phosphorylation of PKB/Akt in muscle between LBWT and NBWT groups. Thus, it appears that downregulation of MSTN signaling pathway was likely a compensatory response.

## Conclusion

In conclusion, LBWT skeletal muscle exhibited reduced abundance and phosphorylation of key components of translation initiation pathway. The lower abundance of 4EBP1 and MNKs did not produce the expected reduction in eIF4E phosphorylation. However, the lower phosphorylation of S6K1, which was correlated to the lower expression of eIF3, and the lower eIF4G abundance and phosphorylation suggested downregulation of translation initiation signaling in muscle of LBWT pigs. This downregulation likely predispose LBWT pigs to slower rates of protein synthesis. In contrast, IGF-I R expression and MSTN signaling were inversely related to LBWT, indicating they may participate in compensatory responses to counteract the lower IGF-I expression and lower protein synthesis signaling in LBWT neonatal pig muscles.

## Author contributions

YC, SJ, RR, and SE designed the research; YC and SM conducted the research; YC and SE analyzed the data; YC, SJ, RR, and SE. wrote the paper; and SE had primary responsibility for the final content. All authors read and approved the final manuscript.

### Conflict of interest statement

The authors declare that the research was conducted in the absence of any commercial or financial relationships that could be construed as a potential conflict of interest.
